# With a British Field Ambulance in Retreat

**Published:** 1914-11-21

**Authors:** 


### WITH A BRITISH FIELD AMBULANCE IN RETREAT.

### By an Army Medical Officer at the Front.

### THE LATER DAYS OF THE RETREAT.

I have described (The Hospital, October 17, 1914, page 63) the first day of the retreat, as far as the time when the sick were put into the hospital. Our section was then placed on the line of retreat. An officer rode by and as he passed told me he believed there were some wounded in the streets in the other direction. It seemed useless to expose my command to risk unless I was sure that the wounded were there. So leaving the section in charge of a junior officer I went off to explore, accompanied by Corporal Chatting of the R.A.M.C., a very courageous man, who volunteered to go with me.* We went down through the deserted streets, but no one was in them. Everything was silent as the grave, except for the incessant rattle of rifle fire without the town. I decided to go back to the waggons and proceed after the retiring forces. It was now getting slightly dusk. As we went we heard of wounded men lying in the streets to the left hand, so we went down and got them on to ambulance waggons. Some we heard of we could not reach because our own guns were firing over us between us and them. I found some men of the Belgian Red Cross Society and managed to explain the situation to them. These men promised to collect the wounded when our guns withdrew. So we went on after the Army.

### A Night March and no Escort.

A little way further on and we soon came up with many baggage waggons and straggling troops in a narrow road. We could not get by, but after some delay they assembled the armed men on the left of the road and passed on the waggons. I found several men singly and in groups straggling along. These I ordered to follow my section, and when I came on larger bodies of their regiment I detached them and handed them over. I had turned southwards in the direction of B—— because I knew it was the direction of probable retirement, and because I heard of some wounded in a village on that road. It had become quite dark now. There was no light excepting the glare of the houses and farms the Germans had set on fire. I passed a village, but on the other side found the way blocked by a lot of waggons reversing. The roads here are often paved with cobble-stones, in consequence the rattle made by these great waggons, many with six horses, was deafening. I could do nothing till the last was round. Then I rode along the column, it was nearly half a mile long, to find the officer in charge. I found him and learnt that this was a divisional ammunition column that had been coming up from the rear and had not heard of our reverse till it had come to the village I had just passed through. I told the officer the position of affairs. He said, "Good God, I have got tons of ammunition here and no escort." We talked over the position. I told him I thought that we were safe till morning from anything except a cavalry raid as I was sure the German infantry must be exhausted by marching and fighting and that we must be holding them back. He had an electric torch. By its light we examined a farmhouse with large store barns attached. Like many farms on the Belgian frontier the barns had immensely thick walls and the walls loop-holed.

### With the Wounded in a Barn.

We then discussed the advisability of stopping there, parking the waggons, and holding off a possible cavalry raid with the divisions' rifles. Just then we saw the light of a motor-car coming down the road, so we stopped it and found it contained a staff officer I knew well at Tidworth. He directed the ammunition column to the best place for it and sent me on to the Headquarters of General Sir H. Smith-Dorrien. After much marching I got there at 12.30 a.m., and saw the General and the Director of Medical Services. We were given a barn in which to rest, wounded and sound all together. It was but a short night, for at 3.15 a.m. we were told to go on to B—— and put the wounded in hospital there. So tired horses and tired men turned out again and off we went. We reached B—— that morning, put the sick into a civilian hospital, and went ourselves into a private house and there washed, ate, and lay down to sleep a while.

At B—— the other sections of No. 7 rolled up later than we did. We had but little rest, for early in the afternoon we got orders to retire out of the town as it was likely to be shelled soon. We could hear the guns to the north of us. Colonel K. ordered me to proceed about six miles down a certain road and await him there. I got there as it got dark, and then had an order from him to come back and camp near him in a field of oats. A lot of the oats were given to the horses, and I turned in by the side of a waggon wheel about 11 p.m. At 3.30 a.m. I was called to read a message—orders—which had just come. It was from the Colonel, telling me we were to move at once on the way we had gone the day before.

### A Chateau and an English Governess.

He had given me a route in the orders. So soon as we were ready I got off, and, as I had foreseen, soon began to get more or less mixed with other troops. But after a while the route diverged to a place called P——, and I was at peace, except for a field ambulance which was ahead of me. After a mile or two I read my map that I should turn sharp to the right, but the other field ambulance kept straight on. I sent an officer after it to tell the officer in charge I thought he was going wrong. By the map I thought he would go into a great forest. He declined to take any notice of my

* Corporal Chatting was later mentioned in dispatches. See The Hospital, October 24, page 82.

suggestion, and I was not going to follow any lead against my own convictions, so he kept his way; I turned mine. I do not know what became of that field ambulance. I was right. We came upon a lovely old château, with moat and drawbridge, woods and waters, and lovely gardens. Here we halted, fed and watered the horses, and let the men breakfast. We found an English girl in some distress of mind but pretty cheerful. She was governess in the family of the noble who owned this château. She had been with the family at another place, but as the Germans came the Count had sent her and other servants to this château. He and his lady had gone off in a motor-car to join a Red Cross Association to which they belonged. And now the Germans were coming this way. I advised them to go to Le Q——, the nearest town, and get the Mayor to send them on to a safer place.

A Fight in the Air.

We ate our breakfast and then continued on our way. We came in again on to the road of the main retirement and joined in it. A vast column of troops of all kinds was moving steadily along. Guns of our rear-guard were pounding away behind us to keep the Germans back, and German aeroplanes flying over us to find our position and take back information. Then I saw a most exciting scene. Our aeroplanes came flying over and began to hunt the Germans. I had a splendid view of one hunt. Two of ours got after a German. They soared above him like hawks above a heron. The German manœuvred to keep high, but they forced him down and forced him down. Then came the order, "All men with rifles to the side of the road"; and they began to "pot" at him. Still he was too high, but our 'planes forced him down lower, and at last the rifles got a bullet into his tank and down he came, gliding like a pricked pheasant, and we marked him land beyond some trees. I was disappointed; I wanted to see the brute get it right in the engine and come down with a crash. The pilot was not hurt and bolted into a wood; we hurt the 'plane though, and I hope the French Territorials had the man later. Another 'plane we did bring down with a crash, but I did not see that. There is no doubt, I believe, that the Germans have been dressing in khaki, putting on Red Cross armlets, and so working surprises on our men.

